# A randomized, double-blind, multicenter, phase III study on the efficacy and safety of a combination treatment involving fimasartan, amlodipine, rosuvastatin in patients with essential hypertension and dyslipidemia who fail to respond adequately to fimasartan monotherapy

**DOI:** 10.1186/s40885-022-00223-4

**Published:** 2022-12-01

**Authors:** Eun-Seok Jeon, Sang Wook Lim, Seok-Yeon Kim, Hyoung-Mo Yang, Moo Hyun Kim, Moo-Yong Rhee, Seung Hwan Han, Jinho Shin, Kwang-il Kim, Jin-Ok Jeong, Ki Chul Sung, Geu Ru Hong, Hyung-Seop Kim, Kihwan Kwon, Tae-Soo Kang, Hae-Young Lee, Su-Eun Han

**Affiliations:** 1 Division of Cardiology, Samsung Medical Center, Sungkyunkwan University School of Medicine, Seoul, Korea; 2 Division of Cardiology, Bundang Cha Hospital, Cha University, Seongnam, Republic of Korea; 3 Department of Cardiology, Cardiovascular Center, Seoul Medical Center, Seoul, Republic of Korea; 4 Division of Cardiology, Ajou University Hospital, Suwon, Republic of Korea; 5 Department of Cardiology, Dong-A University Hospital, Busan, Republic of Korea; 6 Cardiovascular Center, Dongguk University Ilsan Hospital, Goyang, Republic of Korea; 7 Division of Cardiology, Gachon University Gil Medical Center, Incheon, Republic of Korea; 8 Department of Internal Medicine, Hanyang University Hospital, Seoul, Republic of Korea; 9 Department of Internal Medicine, Seoul National University Bundang Hospital, Seongnam, Republic of Korea; 10 Department of Internal Medicine, Chungnam National University Hospital, Daejeon, Republic of Korea; 11 Division of Cardiology, Kangbuk Samsung Hospital, Seoul, Republic of Korea; 12 Division of Cardiology, Yonsei University College of Medicine, Seoul, Republic of Korea; 13 Department of Internal Medicine, Dongsan Medical Center, Keimyung University, Deagu, Republic of Korea; 14 Division of Cardiology, Ewha Womans University College of Medicine, Seoul, Republic of Korea; 15 Division of Cardiology, Dankook Unversity Hospital, Cheonan, Republic of Korea; 16 Department of Internal Medicine, Seoul National University Hospital, Seoul, Republic of Korea; 17 Clinical Strategy Department, Boryung Corporation, 136 Changgyeonggung-ro, Jongno-gu, Seoul, Republic of Korea

**Keywords:** Hypertension, Dyslipidemias, Fimasartan, Amlodipine, Rosuvastatin, Combination drug therapy

## Abstract

**Background:**

To assess the efficacy and safety of a combination therapy involving fimasartan, amlodipine, and rosuvastatin in patients with essential hypertension and dyslipidemia who fail to respond to fimasartan monotherapy.

**Methods:**

This phase III, randomized, double-blind, multicenter study was conducted in adults aged 19–70 years. Patients who voluntarily consented were screened for eligibility to enroll in the study. Patients who failed to respond to 4 weeks of fimasartan monotherapy were randomized with a 1:1:1 ratio to the fimasartan 60 mg/amlodipine 10 mg + rosuvastatin 20 mg (FMS/ALD + RSV) as study group, fimasartan 60 mg/amlodipine 10 mg (FMS/ALD) as control 1 group, and fimasartan 60 mg + rosuvastatin 20 mg (FMS + RSV) as control 2 group. The primary efficacy endpoints were the change in the sitting systolic blood pressure and the rate of change in the low-density lipoprotein cholesterol (LDL-C) level from baseline to 8 weeks. The adverse events, adverse drug reactions, physical examination findings, laboratory test results, electrocardiograms, and vital signs were evaluated to assess safety in the study.

**Results:**

Of 138 randomized patients, 131 were conducted efficacy analysis, and 125 completed the study. For the change in LDL-C and sitting SBP (SiSBP) as primary efficacy assessments, the change in LDL-C at week 8 was significantly reduce in the FMS/ALD + RSV group than in the control 1 group (*P* < 0.001). The change in SiSBP at week 8 were greater reduce in the FMS/ALD + RSV group than in the FMS + RSV group (both *P* < 0.001). For the safety evaluation, there were no differences among the treatment groups in the incidence of adverse drug reactions.

**Conclusions:**

The fimasartan/amlodipine + rosuvastatin combination therapy can effectively and safely lower blood pressure and improve lipid levels in patients with essential hypertension and dyslipidemia who fail to respond adequately to fimasartan monotherapy.

**Trial registration:**

NCT03156842, Registered 17 May 2017

## Background

Studies have shown that a combination therapy using two or more drugs with complementary mechanisms is more effective than a single treatment and can also increase patients’ tolerance to adverse events (AEs) that are dose dependent. Many studies have confirmed that the simultaneous use of calcium channel blockers (CCBs) and angiotensin receptor blockers (ARBs) appears effective [1–3].

Fimasartan is an ARB-based drug that selectively blocks the angiotensin II receptor, especially the AT1 receptor. ARB-based drugs have several advantages and are considered the primary prescription drug for the treatment of hypertension. First, the safety of these drugs is well established compared to that of other types of antihypertensive drugs. In addition, several ARB clinical trials have confirmed that ARBs are clinically efficacious in treating patients with heart failure, myocardial infarction, atrial fibrillation, and diabetic renal impairment as well as improving blood pressure conditions [4, 5]. Moreover, among the many advantages of ARB-based drugs, they also selectively act on the angiotensin II receptor.

CCB-based drugs, including amlodipine, are known to induce cardioprotective effects and treat atherosclerosis as well as reduce blood pressure. Amlodipine is a dihydropyridine-based CCB that mainly acts on vascular smooth muscle cells or L-type (L-type: long lasting) calcium receptors of the myocardium, blocking the influx of calcium into the cells and reducing the resistance of peripheral blood vessels, thereby lowering blood pressure. ARB-type drugs and CCB-type drugs are administered together because they decrease blood pressure even more when administered together compared with alone and have few side effects, so this combination drug is already being developed [6].

Rosuvastatin, like other statin drugs, is known to reduce the concentration of total cholesterol (TC) and LDL-C by inhibiting the enzyme HMG-CoA reductase, an enzyme in the mevalonate pathway, a cholesterol synthesis pathway. In particular, rosuvastatin has the best inhibitory effect of this enzyme due to its high affinity with HMG-CoA reductase, effectively reducing LDL cholesterol levels and increasing HDL cholesterol levels [7].

Based on the facts mentioned above, we intend to develop a combination therapy involving fimasartan, amlodipine, and rosuvastatin, which are already commercially available drugs for hypertension and dyslipidemia. The purpose of this study was to evaluate the efficacy and safety of the fimasartan, amlodipine, and rosuvastatin combination therapy in patients with essential hypertension and dyslipidemia who did not respond adequately to fimasartan monotherapy.

## Methods

### Study design and protocol

**Fig. 1:**
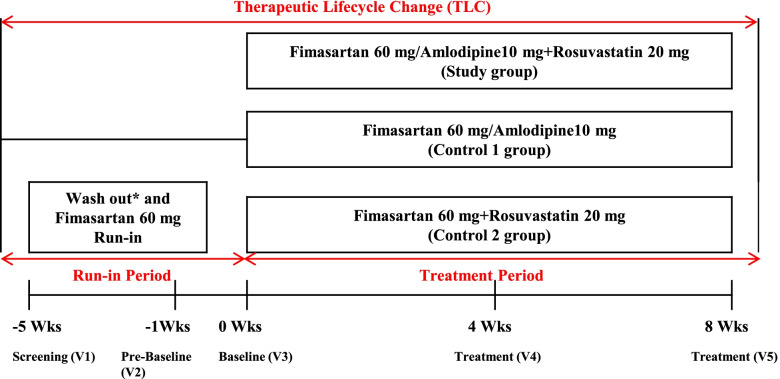
Study design. *In the case of taking fibrate class drugs, at least two weeks of a wash-out period had taken during the screening period before starting the run-in period

This phase III clinical trial was a randomized, double-blind, multicenter study according to the Declaration of Helsinki and Good Clinical Practice guidelines. The institutional review board at each clinical site approved the study. This clinical study targeted males and females aged 19 to 70 years who had were confirmed to have essential hypertension and dyslipidemia at the screening visit (visit 1). If an individual was selected as an eligible patient after the screening test, he or she underwent therapeutic lifestyle changes (TLCs) for 4 weeks (± 5 days) before the prebaseline visit (visit 2), and the TLCs were continued throughout the study period (Fig. 1). Following fimasartan 60 mg monotherapy, patients with uncontrolled blood pressure (140 mmHg ≤ sitting systolic blood pressure [SiSBP] < 180 mmHg) at the baseline visit (visit 3) were recruited.

By the time of the screening examination, the patients who were previously taking antihypertensive/lipid control drugs had undergone a wash-out period of 4 weeks before the baseline visit during the run-in period.

At the baseline visit, the patients who were finally eligible to participate in the study were randomly assigned to the fimasartan 60 mg/amlodipine 10 mg + rosuvastatin 20 mg (FMS/ALD + RSV) as study group, fimasartan 60 mg/amlodipine 10 mg (FMS/ALD) as control 1 group, or fimasartan 60 mg + rosuvastatin 20 mg (FMS + RSV) as control 2 group according to an allocation ratio of 1:1:1. The patients orally consumed three tablets once a day for 8 weeks and visited the clinical sites at week 4 (visit 4) and week 8 (visit 5) for safety and efficacy assessments. To guarantee data quality, monitoring was performed according to the Korean Good Clinical Practice guidelines, and the clinical trial data were collected using an electronic data capture system. The first patient was enrolled on July 31, 2017, and the last patient follow-up was completed on December 7, 2018.

### Patients

Patients (age 19–70 years) with hypertension (140/90 mmHg on antihypertensive medication) and dyslipidemia (defined in accordance with the National Cholesterol Education Program Adult Panel III [NCEP-ATP III] [8] or currently on lipid modifying medications) were included (Table 1). Exclusion criteria were a SiSBP ≥ 180 mmHg at screening and baseline visit and/or sitting diastolic blood pressure (SiDBP) ≥ 110 mmHg; differences between arms ≥ 20 mmHg for SiSBP or ≥ 10 mmHg for SiDBP; secondary hypertension; secondary dyslipidemia (nephrotic syndrome, dysproteinemia, Cushing syndrome, and obstructive hepatopathy); fasting triglyceride level at baseline visit ≥ 400 mg/dL; history of myopathy, rhabdomyolysis, and/or creatinine kinase  ≥ 2-fold upper limit of normal; history of hypersensitivity to angiotensin receptor antagonist and/or HMG-CoA reductase inhibitors; gastrointestinal surgery or active inflammatory gastrointestinal diseases potentially affecting study drug absorption in the preceding 12 months; uncontrolled (glycated hemoglobin > 9% at prebaseline visit) or insulin-dependent diabetes mellitus; liver disease (aspartate aminotransferase and/or alanine aminotransferase  ≥ 2-fold upper normal limit); hepatitis B (including positive test for HBsAg) or hepatitis C-positive; impaired function of kidney (Creatinine clearance < 30 mL/min; human immunodeficiency virus infection; electrolyte imbalance (sodium level < 130 mmol/L or potassium level < 3.5 mmol/L or ≥ 5.5 mmol/L); retinal hemorrhage; visual disturbance or retinal microaneurysm within the past 6 months; history of abusing drugs or alcohol; ischemic heart disease within the previous 6 months (angina pectoris, acute myocardial infarction); peripheral vascular disease); percutaneous coronary intervention, or coronary artery bypass graft within the previous 6 months; severe cerebrovascular disease within previous 6 months (cerebral infarction, or cerebral hemorrhage); New York Heart Association functional class III and VI heart failure; clinically significant cardiac arrhythmia; or history of any type of malignancy within the previous 5 years; women in pregnancy, breastfeeding, or child-bearing potential without no intention of using a contraceptive.

**Table 1:** Inclusion criteria and LDL-C goals for therapeutic lifestyle changes

Risk category	Major risk factors^a^	LDL-C goal (mg/dL)	LDL-C level (mg/dL)	TG level (mg/dL)
High risk	CHD^b^ or CHD risk equivalents^c^ (10-yr risk > 20%)	< 100	≥ 100	< 400
Moderate risk	2 + Risk factors (10 yr risk ≤ 20%)	< 130	≥ 130	
Low risk	0–1 Risk factor	< 160	≥ 160	

*LDL-C* Low-density lipoprotein cholesterol, *TG* Triglyceride, *CHD* Coronary heart disease, *HDL* High-density lipoprotein

^a^ Risk factors: include cigarette smoking, hypertension (blood press ≥ 140/90 mmHg or on antihypertensive medication), low HDL cholesterol (< 40 mg/dL), family history of premature CHD (CHD in male first-degree relative < 55 years of age; CHD in female first-degree relative < 65 years of age), and age (men ≥ 45 years; women ≥ 55 years)

^b^ CHD includes history of myocardial infarction, unstable angina, stable angina, coronary artery procedures (angioplasty or bypass surgery), or evidence of clinically significant myocardial ischemia

^c^ CHD risk equivalents include clinical manifestations of noncoronary forms of atherosclerotic disease (peripheral arterial disease, abdominal aortic aneurysm, and carotid artery disease [transient ischemic attacks or stroke of carotid origin or > 50% obstruction of a carotid artery]), diabetes, and 2 + risk factors with 10-year risk for hard CHD > 20%

### Efficacy and safety evaluation

To assess efficacy, lipid tests targeting the triglyceride (TG), TC, LDL-C, and HDL-C levels were conducted. For blood pressure (SiSBP, SiDBP), the reference arm was selected at the screening visit, and the mean value of three measurements was used.

The primary efficacy assessments were the percentage change in LDL-C after 8 weeks from the baseline, which was compared between the FMS/ALD + RSV group and FMS/ALD group, and the change in SiSBP after 8 weeks from the baseline, which was compared between the FMS/ALD + RSV group and FMS + RSV group.

The secondary efficacy assessments were the change in SiSBP after 8 weeks from the baseline, which was compared between the FMS/ALD + RSV group and FMS/ALD group, and the change in LDL-C after 8 weeks from the baseline, which was compared between the FMS/ALD + RSV group and FMS + RSV group. Additionally, the changes in TC, HDL-C, TG after 8 weeks from the baseline; the blood pressure control rate (SiSBP < 140 mmHg and SiDBP < 90 mmHg) after 8 weeks of treatment; and the ratio of achieving treatment goals according to the NCEP-ATP III guidelines after 8 weeks of treatment (Table 1). The overall compliance of the investigational drug was also evaluated for the duration of the study.

The safety assessments were conducted by AEs, adverse drug reactions (ADRs), vital signs, laboratory test results, physical examination findings, electrocardiograms, chest X-rays, and pregnancy test for the duration of the study.

### Determination of sample size

This phase III study was to demonstrate the superiority of FMS/ALD + RSV group regarding the therapeutic effect measured by the change in SiSBP compared to FMS + RSV group at week 8 from baseline and percentage change in LDL-C compared to FMS/ALD group at week 8 from baseline.

Two statistical hypotheses were therefore established and the subject number was determined. Total test power was set to 80% for the whole hypothesis whereas the two-sided significance level was set to 5% for each hypothesis. Without adjusting multiplicity, each of the hypotheses had the test power set to 90%.

For the change in SiSBP from FMS/ALD + RSV group, the value was assumed to be equal to the change in SiSBP in the FMS + RSV group based on the results of a previous study. For the estimation of the differences in the mean and the standard deviation (SD) between the two groups, weighted mean and pooled SD from the previous studies were utilized [9, 10]. The difference between the two treatment groups in SiSBP response was 13.63 mmHg with a pooled SD of 17.80 mmHg, and it was determined that at least 36 patients were required for each group. Assuming a dropout rate of 20%, at least 135 patients (45 patients per group for three groups) were concluded for the sample size.

For LDL-C, the percent change in LDL-C was –52.35% for FMS + RSV and –6.53% for fimasartan. The difference in the mean was 45.82% and SD was conservatively assumed to be 16.82% through previous study [10]. At least four patients for each group were required as the sample size to compare the LDL-C responses, which results in a total of 12 patients in three groups when dropout rate was assumed as 20%.

In conclusion, as the number of patients required to compare the SiSBP reduces was greater than the number required for the LDL-C reduces, the sample size was determined to be 135 patients.

### Statistical analysis

This study followed the intention-to-treat principle, demographic, baseline data and efficacy assessment was used as the full analysis set (FAS). The safety assessment was performed with the safety set (SS).

The demographic data and baseline characteristics were summarized for each treatment group, and any missing data were analyzed without additional corrections. For the continuous data, descriptive statistics are presented, and the categorical data are presented as frequencies and ratios (%). For the efficacy assessments, analysis of covariance (ANCOVA) was performed using the baseline values as covariates to assess the differences between the two FMS/ALD + RSV groups with a two-sided significance level of 5%. Moreover, the least square (LS) mean and standard error for each administration group, the LS means for the difference between the two groups and the *P*-value and 95% two-sided confidence interval (CI) for both groups are presented.

All AEs were analyzed using MedDRA ver. 21.1 (https://www.meddra.org/) as standardized data for the “system organ class” and “preferred term.” The percentage of patients who experienced any AEs between groups was compared using chi-square or Fisher exact tests. Incidence of AEs was presented according to relationship with study drugs. All statistical analyses were performed using SAS ver. 9.3 (SAS Institute, Cary, NC, USA).

## Results

### Disposition of the patients

**Fig. 2:**
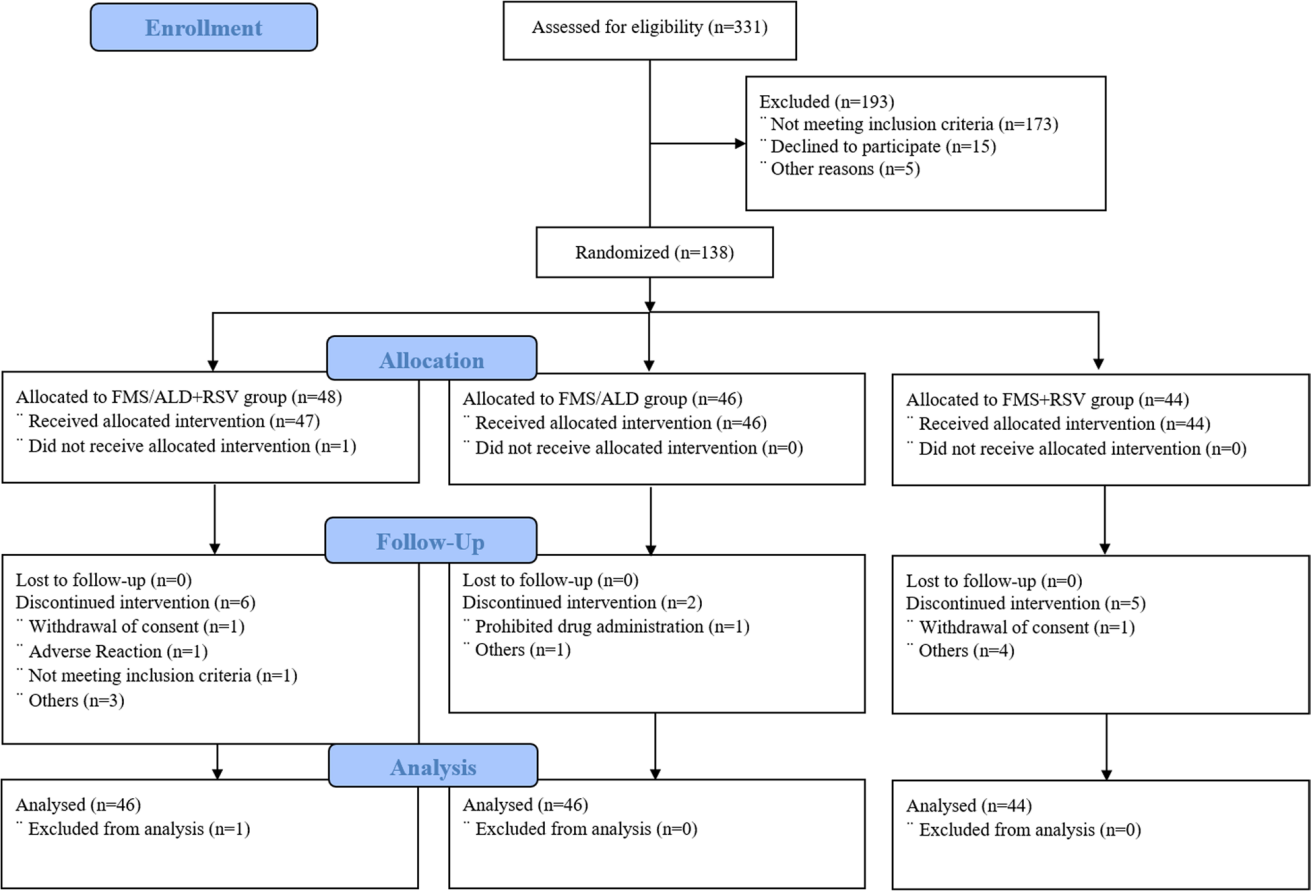
Flow diagram., FMS/ALD + RSV group, fimasartan 60 mg/amlodipine 10 mg + rosuvastatin 20 mg treatment, study group; FMS/ALD group, fimasartan 60 mg/amlodipine 10 mg treatment, control 1 group; FMS + RSV group, fimasartan 60 mg + rosuvastatin 20 mg treatment, control 2 group, FAS, full analysis set; PPS, per protocol set; SS, safety set

A total of 331 patients from the 26 sites were screened, and 193 patients who did not meet the inclusion and exclusion criteria in the eligibility assessment were excluded. Accordingly, 138 patients were randomly assigned and 125 patients completed the clinical trial. For the reasons for dropout, withdrawal of consent (*n* = 2), ADR (*n* = 6), deviation of the inclusion and exclusion criteria (*n* = 2) and other reason (*n* = 3) were noted. Of the 138 patients randomly assigned to groups in this clinical trial, 131 patients were included in efficacy analysis after excluding seven patients owing to missing efficacy data (Fig. 2).

### Demographics and baseline characteristics

The mean ± SD age of the patients was 60.42 ± 6.83 years; 85 patients (64.89%) were aged 19 − 64 years, and 46 patients (35.11%) were aged 65 years or older. The baseline SiSBP and SiDBP were 153.14 ± 9.15 mmHg and 91.59 ± 8.26 mmHg, respectively. The baseline LDL-C and HDL-C were 155.40 ± 32.35 mg/dL and 47.71 ± 12.15 mg/dL, respectively. The demographics and baseline characteristics were not significantly different between the groups (Table 2).

**Table 2:** Demographics and baseline characteristics (full analysis set)

Characteristic	FMS/ALD + RSV^a^ (*n* = 43)	FMS/ALD^b^ (*n* = 45)	FMS + RSV^c^ (*n* = 43)	*P*-value^d^
**Demographic**				
Age (yr)	59.65 ± 7.43	60.84 ± 6.29	60.74 ± 6.85	0.743
Age group (yr)				0.631
19–64	30 ± 69.77	27 ± 60.00	28 ± 65.12	
≥ 65	13 ± 30.23	18 ± 40.00	15 ± 34.88	
Sex				0.542
Male	32 (74.42)	36 (80.00)	30 (69.77)	
Female	11 (25.58)	9 (20.00)	13 (30.23)	
Weight (kg)	73.14 ± 12.70	71.29 ± 10.01	74.53 ± 11.52	0.335
Height (cm)	164.87 ± 8.08	164.84 ± 7.67	164.81 ± 8.37	0.999
BMI (kg/m^2^)				0.202
≥ 18.5 to < 23	3 (6.98)	7 (15.56)	1 (2.33)	
≥ 23 to < 25	7 (16.28)	10 (22.22)	8 (18.60)	
≥ 25 to < 30	28 (65.12)	25 (55.56)	25 (58.14)	
≥ 30	5 (11.63)	3 (6.67)	9 (20.93)	
Smoke				0.548
Smoker	11 (25.58)	12 (26.67)	12 (27.91)	
Nonsmoker	17 (39.53)	17 (37.78)	22 (51.16)	
Ex-smoker	15 (34.88)	16 (35.56)	9 (20.93)	
Drink				0.385
Drinker	26 (60.47)	26 (57.78)	20 (46.51)	
Nondrinker	17 (39.53)	19 (42.22)	23 (53.49)	
**Baseline characteristic**				
SiSBP (mmHg)	153.98 ± 10.29	152.93 ± 8.69	152.52 ± 8.56	0.912
SiDBP (mmHg)	89.71 ± 8.01	90.69 ± 8.61	94.41 ± 7.53	0.019
LDL-C (mg/dL)	157.21 ± 27.45	151.22 ± 32.86	157.95 ± 36.43	0.566
TC (mg/dL)	221.77 ± 28.38	213.51 ± 35.56	223.77 ± 40.14	0.348
TG (mg/dL)	196.42 ± 79.24	180.53 ± 63.86	188.40 ± 75.13	0.769
HDL-C (mg/dL)	48.44 ± 13.65	45.93 ± 12.07	48.84 ± 10.59	0.361
Pulse (beats/min)	74.65 ± 11.83	77.51 ± 12.69	77.23 ± 11.35	0.619

Data are presented as mean ± standard deviation or number (%)

*BMI* Body mass index, *SiSBP* Sitting systolic blood pressure, *SiDBP* Sitting diastolic blood pressure, *LDL-C* Low-density lipoprotein cholesterol, *TC* Total cholesterol, *TG* Triglyceride, *HDL-C* High-density lipoprotein cholesterol

^a^ FMS/ALD + RSV; study group, fimasartan 60 mg/amlodipine 10 mg + rosuvastatin 20 mg treatment

^b^ FMS/ALD; control 1 group, fimasartan 60 mg/amlodipine 10 mg treatment

^c^ FMS + RSV; control 2 group, fimasartan 60 mg + rosuvastatin 20 mg treatment

^d^ Statistical methods testing for difference among treatment groups (ANOVA, Kruskal–Wallis test, chi-square test, or Fisher exact test)

### Clinical efficacy

The percentage change of LDL-C from baseline at week 8 showed greater in the FMS/ALD + RSV group than in the FMS/ALD group (–48.52 ± 18.63 vs. 4.43 ± 18.27, *P* < 0.001). The percentage change of LDL-C between the FMS/ALD + RSV group and FMS + RSV group was not different (–48.57 ± 2.77 vs. –49.64 ± 2.77, *P* = 0.785). The least square mean (LSM) difference of LDL-C percentage change between FMS/ALD + RSV group and FMS/ALD group treatment groups was –43.69% ± 3.95% (95% CI, –51.55 to –35.83).

**Fig. 3:**
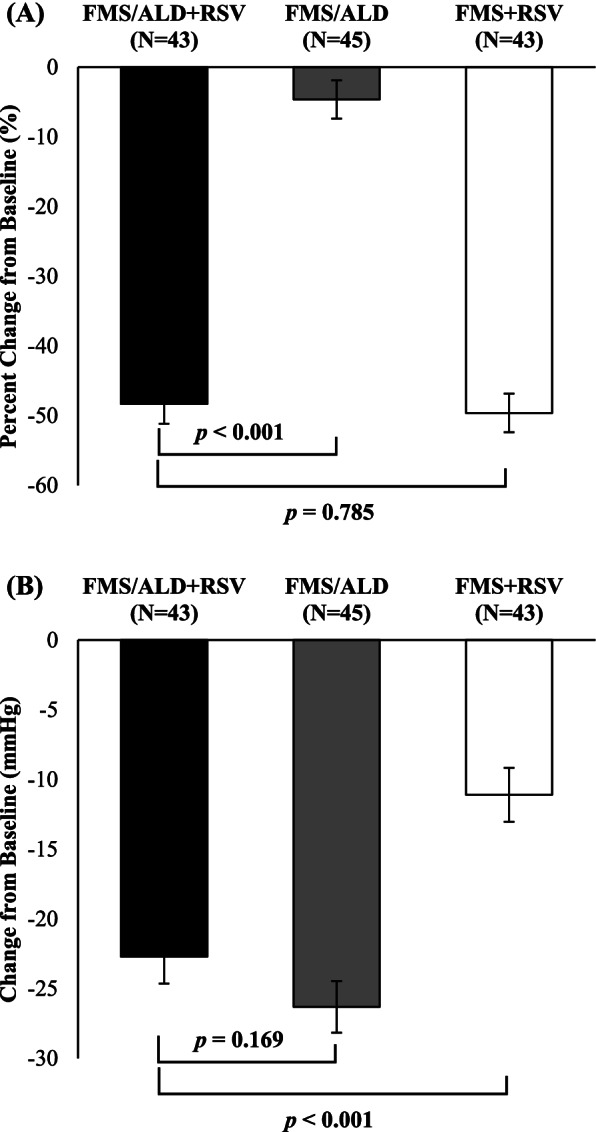
Reduction of low-density lipoprotein cholesterol (LDL-C) and sitting systolic blood pressure (SiSBP) from baseline at week 8. **A** Percent change from baseline in LDL-C. **B** Change from baseline in SiSBP

The FMS/ALD + RSV group was significantly reduce in SiSBP from baseline at week 8 compared to that reported for FMS + RSV group (–22.72 ± 1.93 vs. –11.11 ± 1.93, *P* < 0.001). The changes of SiSBP was not significantly different between FMS/ALD + RSV group and FMS/ALD groups (–22.63 ± 1.90 vs. –26.32 ± 1.85, *P* = 0.169). Likewise, the reduction in SiDBP from baseline at week 8 was significantly larger in the FMS/ALD + RSV group compared to that in the FMS + RSV group (–12.71 ± 1.23 vs. –6.94 ± 1.23, *P* = 0.002). The FMS/ALD + RSV group and FMS/ALD group were not significantly different in the reduction of SiDBP (–11.83 ± 1.08 vs. –12.88 ± 1.06, *P* = 0.490). The LSM difference in SiSBP and SiDBP between FMS/ALD + RSV group and FMS + RSV group was − 11.62 mmHg (95% CI, − 17.04 to − 6.19) and − 5.78 mmHg (95% CI, − 9.30 to − 2.25), respectively. Changes in SiSBP, SiDBP and LDL-C are presented in Table 3 and Fig. 3.

**Table 3:** Change in SiSBP, SiDBP, and LDL-C from baseline at week 8

Variable	Treatment groups				FMS/ALD + RSV vs. FMS/ALD		FMS/ALD + RSV vs. FMS + RSV	
	FMS/ALD + RSV^a^ (*n* = 43)	FMS/ALD^b^ (*n* = 45)	FMS + RSV^c^ (*n* = 43)	LSM difference (SE)		*P*-value^d^	LSM difference (SE)	*P*-value^d^
SiSBP (mmHg)								
Baseline	153.98 ± 10.29	152.93 ± 8.69	152.52 ± 8.56					
Week 8	131.00 ± 13.77	126.94 ± 11.84	141.67 ± 14.18					
Change	–22.98 ± 13.65	–25.99 ± 13.87	–10.85 ± 12.24					
*P*-value	< 0.001	< 0.001	< 0.001					
ANCOVA result								
LSM (SE)	–22.72 ± 1.93	–26.32 ± 1.85	–11.11 ± 1.93	3.68 ± 2.65		0.169	–11.62 ± 2.73	< 0.001
SiDBP (mmHg)								
Baseline	89.71 ± 8.01	90.69 ± 8.61	94.41 ± 7.53					
Week 8	78.17 ± 8.68	77.53 ± 6.98	86.31 ± 8.79					
Change	–11.54 ± 8.86	–13.16 ± 8.21	–8.10 ± 8.57					
*P*-value	< 0.001	< 0.001	< 0.001					
ANCOVA result								
LSM (SE)	–11.83 ± 1.08	–12.88 ± 1.06	–6.94 ± 1.23	1.05 ± 1.51		0.490	–5.78 ± 1.77	0.002
LDL-C (mg/dL)								
Baseline	157.21 ± 27.45	151.22 ± 32.86	157.95 ± 36.43					
Week 8	80.67 ± 32.29	143.58 ± 36.70	77.42 ± 27.26					
Percent change	–48.52 ± 18.63	–4.43 ± 18.27	–49.68 ± 18.19					
*P*-value	< 0.001	0.111	< 0.001					
ANCOVA result								
LSM (SE)	–48.32 ± 2.82	–4.63 ± 2.76	–49.64 ± 2.77	–43.69 ± 3.95		< 0.001	1.07 ± 3.91	0.785

Data are presented as mean ± standard deviation unless otherwise indicated

*SiSBP* Sitting systolic blood pressure, *SiDBP* Sitting diastolic blood pressure, *LDL-C* Low-density lipoprotein cholesterol, *LSM* Least square mean, *SE* Standard error, *ANCOVA* Analysis of covariance

^a^ FMS/ALD + RSV; Study group, fimasartan 60 mg/amlodipine 10 mg + rosuvastatin 20 mg treatment

^b^ FMS/ALD; Control 1 group, fimasartan 60 mg/amlodipine 10 mg treatment

^c^ FMS + RSV; Control 2 group, fimasartan 60 mg + rosuvastatin 20 mg treatment

^d^ Comparison between treatment was analyzed by ANCOVA model adjusted for factor and baseline values

**Fig. 4:**
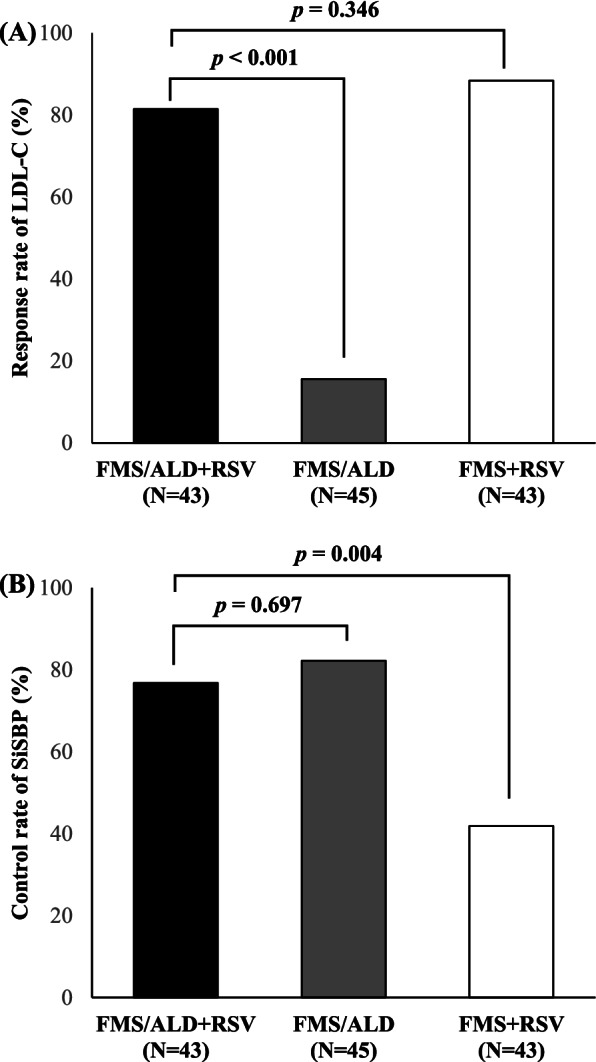
Response rate of low-density lipoprotein cholesterol (LDL-C) and control rate of sitting systolic blood pressure (SiSBP) from baseline at week 8. **A** Response rate of LDL-C. **B** Control rate of SiSBP

The control rate of BP and response rate of LDL-C at week 8 are presented in Fig. 4. The control rate in FMS/ALD + RSV group, FMS/ALD group, and FMS + RSV group was 76.74, 82.22, and 41.86%, respectively (FMS/ALD + RSV vs. FMS/ALD; odds ratio [OR], 0.80; *P* = 0.697). The response rate of LDL-C in FMS/ALD + RSV group, FMS/ALD group, and FMS + RSV group was 81.40, 15.56, and 88.37%, respectively (FMS/ALD + RSV vs. FMS + RSV; OR, 0.56; *P* = 0.346) (Fig. 4).

Comparable to changes in LDL-C, FMS/ALD + RSV group also showed a greater lowering effect on TC and TG, as well as HDL-C elevation compared to that reported for FMS + RSV group (Table 4). Overall compliance of treatment period in FMS/ALD + RSV group, FMS/ALD group, and FMS + RSV group was 94.49 ± 8.84, 96.74 ± 6.34, and 92.28 ± 18.36, respectively (*P* = 0.117). There were not significantly different.

**Table 4:** Percent change in total cholesterol, HDL-C, and triglyceride from baseline at week 8

Variable	Treatment groups			FMS/ALD + RSV vs. FMS/ALD		FMS/ALD + RSV vs. FMS + RSV	
	FMS/ALD + RSV^a^ (*n* = 43)	FMS/ALD^b^ (*n* = 45)	FMS + RSV^c^ (*n* = 43)	LSM difference (SE)	*P*-value^d^	LSM difference (SE)	*P*-value^d^
TC (mg/dL)							
Baseline	221.77 ± 28.38	213.51 ± 35.56	223.77 ± 40.14				
Week 8	150.81 ± 33.64	205.91 ± 39.13	144.37 ± 29.51				
Percent change	–31.73 ± 13.93	–3.18 ± 12.76	–34.44 ± 13.35				
*P*-value	< 0.001	0.102	< 0.001				
ANCOVA result							
LSM (SE)	–31.44 ± 2.03	–3.45 ± 1.98	–34.32 ± 1.99	–27.99 ± 2.85	< 0.001	2.46 ± 2.81	0.384
HDL-C (mg/dL)							
Baseline	48.44 ± 13.65	45.93 ± 12.07	48.84 ± 10.59				
Week 8	55.00 ± 16.16	46.53 ± 11.01	53.42 ± 10.41				
Percent change	14.60 ± 18.50	3.32 ± 17.51	11.51 ± 18.84				
*P*-value	< 0.001	0.211	0.002				
ANCOVA result							
LSM (SE)	15.18 ± 2.62	2.77 ± 2.56	11.62 ± 2.68	12.41 ± 3.68	0.001	2.88 ± 3.80	0.450
TG (mg/dL)							
Baseline	196.42 ± 79.24	180.53 ± 63.86	188.40 ± 75.13				
Week 8	161.74 ± 88.30	181.09 ± 77.77	148.14 ± 70.34				
Percent change	–14.84 ± 35.59	7.73 ± 46.90	–15.73 ± 38.11				
*P*-value	0.009	0.629	0.001				
ANCOVA result							
LSM (SE)	–13.30 ± 6.07	6.26 ± 5.94	–16.29 ± 5.42	–19.56 ± 8.52	0.024	2.00 ± 7.67	0.795

Data are presented as mean ± standard deviation unless otherwise indicated

*TC* Total cholesterol, *HDL-C* High-density lipoprotein cholesterol, *TG* Triglyceride, *ANCOVA* Analysis of covariance, *LSM* Least square mean, *SE* Standard error

^a^ FMS/ALD + RSV; study group, fimasartan 60 mg/amlodipine 10 mg + rosuvastatin 20 mg treatment

^b^ FMS/ALD; control 1 group, fimasartan 60 mg/amlodipine 10 mg treatment

^c^ FMS + RSV; control 2 group, fimasartan 60 mg + rosuvastatin 20 mg treatment

^d^ Comparison between treatment was analyzed by ANCOVA model adjusted for factor and baseline values

### Safety and tolerability

The safety analysis was conducted in 136 patients, the incidence of AEs and ADRs were 18.38% and 5.88%, respectively (Table 5). There was no significant difference in the incidence of ADRs among groups (*P *= 0.907). Adverse events reported were oedema peripheral, dizziness, essential tremor, nasopharyngitis, influenza, nausea, blood creatine phosphokinase increased, rash, pruritus (*n *= 1, respectively) in the FMS/ALD + RSV group; oedema peripheral, asthenia, chronic gastritis, musculoskeletal stiffness, hot flush (*n* = 1, respectively) in the FMS/ALD group; headache (*n* = 3), nasopharyngitis (*n* = 2), blood creatine phosphokinase increased (*n* = 2), chest pain, injury associated with device, abdominal pain upper, diarrhoea, blood lactate dehydrogenase increased, rash, arthralgia, clavicle fracture, gastric cancer (*n* = 1, respectively) in the FMS + RSV group.

**Table 5:** Overall summary of AE and ADR

Variable	FMS/ALD + RSV group^a^ (*n* = 46)	FMS/ALD group^b^ (*n* = 46)	FMS + RSV group^c^ (*n* = 44)	Total (*n* = 136)
AE	8 (17.39) [9]	4 (8.70) [5]	13 (29.55) [16]	25 (18.38) [30]
95% CI	6.44–28.34	0.55–16.84	16.06–43.03	11.87–24.89
*P*-value				0.038
Severity (mild, moderate, severe)	8, 1, 0	5, 0,0	14, 1, 1	27, 2, 1
ADR	3 (6.52) [4]	2 (4.35) [3]	3 (6.82) [4]	8 (5.88) [11]
Exact 95% CI	1.37–17.90	0.53–14.84	1.43–18.66	2.57–11.26
*P*-value				0.9074
Severity (mild, moderate, severe)	4, 0, 0	3, 0, 0	4, 0, 0	11, 0, 0
Drug related adverse reactions				
Headache	0	0	2 (4.55) [2]	2 (1.47) [2]
Essential tremor	1 (2.17) [1]	0	0	1 (0.74) [1]
Asthenia	0	1 (2.17) [1]	0	1 (0.74) [1]
Oedema peripheral	0	1 (2.17) [1]	0	1 (0.74) [1]
Blood creatine phosphokinase increased	0	0	1 (2.27) [1]	1 (0.74) [1]
Blood lactate dehydrogenase increased	0	0	1 (2.27) [1]	1 (0.74) [1]
Pruritus	1 (2.17) [1]	0	0	1 (0.74) [1]
Rash	1 (2.17) [1]	0	0	1 (0.74) [1]
Nausea	1 (2.17) [1]	0	0	1 (0.74) [1]
Hot flush	0	1(2.17) [1]	0	1 (0.74) [1]

Data are presented as number of patients (%) and [case]

*AE* Adverse event, *ADR* Adverse drug reaction, *CI* Confidence interval

^a^ FMS/ALD + RSV; study group, fimasartan 60 mg/amlodipine 10 mg + rosuvastatin 20 mg treatment

^b^ FMS/ALD; control 1 group, fimasartan 60 mg/amlodipine 10 mg treatment

^c^ FMS + RSV; control 2 group, fimasartan 60 mg + rosuvastatin 20 mg treatment

The reported ADRs were essential tremor, pruritus, rash, nausea in the FMS/ALD + RSV group (*n* = 1, respectively). There were no AEs or ADRs that resulted in death during the study. Regarding safety evaluation, there was no significant differences among the groups in ADRs, and no specific issues other than known AEs were found.

## Discussion

Currently, many hypertensive patients cannot control their blood pressure with a single drug, and thus, fixed-dose combination drug prescriptions are increasingly being used. In addition to patients who have not responded to existing drugs, patients who require active blood pressure management from the early stage of treatment can be prescribed combination drugs [11, 12]. Combination drugs improve patient compliance [13] and reduces the frequency of visits needed for titration of each drug, which is convenient for patients and lowers their blood pressure more quickly. Moreover, it is known that the proportion of hypertensive patients with dyslipidemia is high. Due to a synergistic effect, the risk of cardio-cerebrovascular disease increases when both diseases exist, and it has been recommended that both diseases be treated simultaneously. Moreover, several studies have demonstrated that the risk of cardio-cerebrovascular disease can be reduced effectively when both diseases are treated simultaneously [14].

This study was evaluated that combination of fimasartan 60 mg/amlodipine 10 mg + rosuvastatin 20 mg for 8 weeks to patients with hypertension and dyslipidemia was effective and safe in lowering blood pressure and LDL-C.

The FMS/ALD + RSV group showed significantly different that SiSBP and SiDBP lowering effects than did the FMS + RSV group. In addition, compared to the FMS/ALD group, the FMS/ALD + RSV group showed significant increase in HDL-C and decreases in LDL-C, TC, and TG.

From these results, it was confirmed that the fimasartan/amlodipine + rosuvastatin therapy lowered blood pressure, improved lipid levels, and significantly reduced both lipid and blood pressure levels more than the control drug did. Additionally, the FMS/ALD + RSV group showed a higher control rate than did the FMS + RSV group and a higher goal attainment rate of LDL-C than did the FMS/ALD group.

In terms of safety, there were no significant differences among groups in the incidence of ADRs. No ADRs that occurred in the study group were different from those described as potential side effects of existing study drugs and fimasartan + rosuvastatin drugs.

Fimasartan is a drug used to treat hypertension that selectively blocks angiotensin II receptor type 1 and its safety and efficacy have been proven in various studies [15]. It is necessary for most patients to receive more than two medications, as monotherapy is often not enough to control BP completely. In addition, combining medications with different action mechanism induces an additive BP-lowering effect through mechanism that complements one another. Furthermore, a treatment method with a fixed-dose can increase patient compliance and convenience by reducing the required number of drugs and visits for the titration of each drug, and by lowering BP in a shorter amount of time [16, 17]. Therefore, for better control of BP, the current guidelines recommend treatment regimens that combine low-dose medications or incorporates a different class of drug at low doses. The most frequently used fixed-dose combination therapy among many combinations of antihypertensive drugs are renin-angiotensin system (RAS) inhibitors and CCBs. On the other hand, patients who takes ARBs have the highest adherence level, followed by angiotensin-converting enzyme (ACE) inhibitors, CCBs, β-blockers, and diuretic agents. The good safety profile of ARB also allows effective treatment of hypertension and heart failure. As a result, fix-dose therapies most commonly combine ARBs instead of ACE inhibitors for inhibition of RAS. Amlodipine is a dihydropyridine calcium antagonist that gradually and sustainably reduces BP by lowering peripheral vascular resistance. When concomitantly used with other antihypertensive agents, amlodipine has the advantage of showing a stronger BP-lowering effect [18]. Additionally, the incidence of amlodipine side effects that depend on dose, such as edema in ankles, are lowered when used concomitantly with an ARB [18]. Rosuvastatin is an HMG-CoA reductase inhibitor that effectively lowers LDL-C more than other members of the statin group and is an effective medication for preventing cardiovascular diseases (CVD) [7]. Between the overall safety profiles of study treatment, fimasartan/amlodipine treatment, and fimasartan + rouvastatin treatment, there were no significant differences in the study results. This study showed that blood pressure lowering and LDL-C reduction was apparent after week 4 and that there was no further reduction in blood pressure and LDL-C between week 4 and 8.

In effect, therapeutic inactivity results from the delay in dose titration and a prior study has indicated that early goal achievement has been proven beneficial in the prevention of CVD in high-risk patients [19]. Consequently, a fixed-dose combination of study, conceivably be an appropriate option for hypertensive and dyslipidemic patients who fail to respond sufficiently to fimasartan monotherapy.

### Limitation

Our study has limitations. First, a larger study population would be better to assess the primary endpoint of SiSBP and LDL-C reduction and target level attainment after 8 weeks with three treatment groups. Second, the ethical issue that FMS/ALD group do not receive anti-dyslipidemia drugs.

## Conclusion

In this study, among the patients with essential hypertension and dyslipidemia who did not respond to fimasartan monotherapy, those who took study had lower blood pressures and improved lipid levels than did those in the FMS/ALD group and FMS + RSV group. Additionally, when the three single drugs (fimasartan 60 mg, amlodipine 10 mg, and rosuvastatin 20 mg), which have been shown to be effective in monotherapy, are prescribed as a fixed-dose combination drug, the improvements in blood pressure and lipid levels may occur earlier, and the frequency of drug titration visits may be reduced for patients’ convenience.

## Data Availability

The datasets used and/or analysed during the current study are available from the corresponding author on reasonable request.
